# Correspondence

**Published:** 1898-09

**Authors:** E. Van Goidtsnoven

**Affiliations:** Atlanta, Ga.


### Correspondence.

*Editor Southern Medical Record:*

So often have I been asked why I took an active part in the protest of the Alumni of the Southern Medical College against the action of the trustees of that institution in fusing with the Atlanta Medical College, that I deem an explanation necessary and beg you will give it publicity in the columns of your valuable and esteemed journal.

By all who know me I believe that it will be conceded that I entertain no ill feeling against any member of either faculty. If I know well my own heart, I love and respect them all.

I am an alumnus of the Southern Medical College, and hold my diploma from that institution. The Southern Medical College is therefore my *Alma Mater.* To any man who understands the import of that term, it is obvious and plain why I was present at the meetings of the alumni and took part in their deliberations. My presence there was a solemn protest against the unwarranted scheme that lately annihilated our college and consigned it to perpetual oblivion. This transaction arrogantly established the fact that the Southern Medical College must die and that the Atlanta Medical College must live and survive as the fittest.

I do not blame the Atlanta College for having absorbed such a flourishing institution as the Southern Medical. It was certainly a most valuable prize to covet. When a set of medical men establish and found a medical college, they assume a great responsibility, for they do not engage simply in a business venture. They create an alma mater which, like the building of a church, involves sacred duties, not only towards students but towards graduates and alumni. To destroy an alma mater is to injure her graduates and jeopardize in a great measure their future usefulness and reputation. There was nothing in the pre-existing conditions and circumstances of the Southern Medical College that called for and demanded such a change.

Precedents might be invoked in justification of this late transaction. The absorption of the Southern Medical College by the Atlanta Medical College has neither precedent nor parallel in the history of collegiate consolidation. The Southern Medical College has suddenly, unexpectedly and completely disappeared through an octopus-like process, and the "*Atlanta College*" is the only sign-board that remains plainly visible over the paunch of the stuffed and inflated survivor.

Had the members of the Southern Medical faculty been graduates of that institution, I venture to say they never would have consented to the perpetration of such a scheme. For I know of no medical man who would stab his alma mater, or fostering mother, any more than he would his natural mother.

Very respectfully,

E. VAN GOIDTSNOVEN, M. D.

Atlanta, Ga., Sept. 2, 1898.

